# Supplementary material for: Chitosan Biosynthesis and Virulence in the Human Fungal Pathogen Cryptococcus gattii
Source: mSphere. 2019 Oct 9;4(5):e00644-19. doi: 10.1128/mSphere.00644-19 (PMC6796976; doi:10.1128/mSphere.00644-19)
Supplement: FIG S1 [file mSphere.00644-19-sf001.pdf]

<u>Alignment of Cda1 sequence (H99) with *C. gatti* (CNBG_1745) Cda1 sequence</u>

```
H99     MFTFAAFSALLISLAGVVAQTTGTSVDSSILTKTADSTGPSGFSIPALSELTSGAPTDST  60
        M  FAA SALL+SLAG++AQT  TSV SS+LT+TA  TGPSGFSIPALSELTSGAPTD+T
R265    MSAFAAVSALLVSLAGIMAQTASTSVASSVLTQTA-PTGPSGFSIPALSELTSGAPTDTT  59

H99     VALYSTFAAGATPTVSGAPVLPTSALTIADYPALDVTPPTNSSLVKDWMAKIDLSKVPSY  120
        VALYSTF A ATPTVSGAPVLPTSALTIA+YPALDV PPTNSSLV++W+AKID++KVP+Y
R265    VALYSTFPASATPTVSGAPVLPTSALTIANYPALDVIPPTNSSLVQEWLAKIDMTKVPNY  119

H99     NVTTGDCSTDAAAISDGRCWWTCGGCTRETDIVECPDKNVWGLSYDDGPSPFTPLLIDYL  180
        N TTGDCSTD  A SDGRCWWTCGGCTR TDIV CPDKNVWGLSYDDGPSPFTPLLIDYL
R265    NATTGDCSTDPGATSDGRCWWTCGGCTRATDIVACPDKNVWGLSYDDGPSPFTPLLIDYL  179

H99     QEKNIKTTFFVVGSRVLSRPEMLQTEYMSGHQISIHTWSHPALTTLTNEEIVAELGWTMK  240
        QEKNIKTTFFVVGSRVLSRPEMLQTEYMSGH+ISIHTWSHPALTTLTNE+IVAEL WTMK
R265    QEKNIKTTFFVVGSRVLSRPEMLQTEYMSGHEISIHTWSHPALTTLTNEQIVAELAWTMK  239

H99     VIKDTLGVTPNTFRPPYGDIDDRVRAIAAQMGLTPVIWTSYTDGSTTVNFDTNDWHISGG  300
        VIKDT+GVTPNTFRPPYGDIDDRVRAIAAQMGLTPVIWTSY+DGSTTVNFDTNDWHISGG
R265    VIKDTIGVTPNTFRPPYGDIDDRVRAIAAQMGLTPVIWTSYSDGSTTVNFDTNDWHISGG  299

H99     TATGASSYETFEKILTEYAPKLDTGFITLEHDIYQQSVDLAVGYILPQVLANGTYQLKSI  360
        TATGASSYETFEKIL EYAPKL+TGFITLEHD+YQQSVDLAVGYILPQVLANGTYQLKSI
R265    TATGASSYETFEKILNEYAPKLNTGFITLEHDLYQQSVDLAVGYILPQVLANGTYQLKSI  359

H99     INCLGKDTSEAYIETSSNQTTTQITAATGSQSTFFQPIVGTATGAEVSAPSEATGSTAAG  420
        INCLGKD SEAYIETSSNQTTTQIT+A+G  ST+FQPIVGTATG+EVSAPSEAT  + A
R265    INCLGKDISEAYIETSSNQTTTQITSASG--STYFQPIVGTATGSEVSAPSEAT-GSTAA  416

H99     SAASTTSGSGASASTGAASNTSSSGSGRSATMGGALIALAAVAVGMVYVA  470
         +A+ ++ + + ++   AS++SSSGSGRSATMGGALIA AAVAVG+VYVA
R265    GSAAGSAATTSGSAASGASDSSSSGSGRSATMGGALIAFAAVAVGIVYVA  466
```

400/470 identical(85%); 435/470 positive (92%)

Alignment of Cda2 sequence (H99) with *C. gatti* (CNBG 0964) Cda2 sequence

```
H99         MIPSTAAALLTLTAGAAFAHTGCGGHEIGRRNVGGPMLYRRAVTDEASAAVSTDINTECT  60
            MIPSTAAA LTLTAG AFAH GCGG EIGRRNVGGPML+ RAVTDEASAAVSTD++TECT
R265        MIPSTAAAFLTLTAGTAFAHIGCGGQEIGRRNVGGPMLHSRAVTDEASAAVSTDVSTECT  60

H99         AYSYAPVTELISSFPTIWQTASIPSNDTEAQQLFGKINSTLNTKIPNDVPHGTPTGDWTG  120
            AY YAPVT++ SSFP IWQTASI S D+EAQQLF  IN+T+N+K+PNDVPHGTPTG+WTG
R265        AYGYAPVTQIASSFPAIWQTASILSTDSEAQQLFASINATVNSKLPNDVPHGTPTGNWTG  120

H99         VNYSNSDPDCWWTHNKCTTPSNDTGLQADISIAPEPMTWGLGFDDGPNCSHNALYDLLLE  180
            V+YS+SDPDCWWTHNKCTTPS+DTGL+ADI+  PEPMTWGLGFDDGPNCSHNALYDLLLE
R265        VSYSSSDPDCWWTHNKCTTPSSDTGLKADITTVPEPMTWGLGFDDGPNCSHNALYDLLLE  180

H99         NNQKATMFFIGSNVLDWPLQAMRAHDEGHEICVHTWSHQYMTALSNEVVFAELYYTQKAI  240
            NNQKATMF+IGSNV+DWPLQAMRAHDEGHEICVHTWSHQYMTALSNEVVFAELYYTQKAI
R265        NNQKATMFYIGSNVMDWPLQAMRAHDEGHEICVHTWSHQYMTALSNEVVFAELYYTQKAI  240

H99         KAVLGVTPQCWRPPYGDVDNRVRMIAEGLNLTTIIWSDDTDDWAAGTNGVTEQDVTNNYQ  300
            KAVLGVTP CWRPPYGDVDNRVRMIA  LNL+TI+WSDDT+DW AGTNGVT+QDVTNNYQ
R265        KAVLGVTPLCWRPPYGDVDNRVRMIAAALNLSTIVWSDDTNDWEAGTNGVTQQDVTNNYQ  300

H99         SVIDKAGNGTYTTHGPVVLNHELTNYTMSVFMTMFPKIKSAFNYIVPICTAYNITQPYAE  360
            SVIDKAGNGTYTTHGPVVLNHELTNYTMSVF++MFPKIKSAF+YIVPICTAYNITQPYAE
R265        SVIDKAGNGTYTTHGPVVLNHELTNYTMSVFVSMFPKIKSAFSYIVPICTAYNITQPYAE  360

H99         SNITCPNFETYISGVTNISSSTTQKDGSSSTNTASGSGAAGSASATSSSDDSSSSGGSSG  420
            SN+TCPNFETYISGVTNIS+STTQKDGSSSTNT+  +  + S SA+S+   +SS
R265        SNVTCPNFETYISGVTNISTSTTQKDGSSSTNTSYTASGSTSPSASSTGKIASS------  414

H99         SSGSNNASSGALGMFDSLSGVGLILGGVVAGVMLL  455
                  A SGALGM+D LSG+GLILGGVVAGVMLL
R265        ------AKSGALGMYDGLSGMGLILGGVVAGVMLL  443
```

79/455 identical (83%); 414/455 positive (90%)

Alignment of Cda3 sequence (H99) with *C. gatti* (CNBG 0806) Cda3 sequence

```
H99      MYGHLSLSALSLFAVVAAAPFRESWLQPRDSPVSQLFRRTAPDPNSNDYMSYYPGPGSTP  60
         MYGHLSLSALSL AVVAAAPF ESWLQPRDSPVSQLFRR APDPN++DY+S+YP PGSTP
R265     MYGHLSLSALSLLAVVAAAPFHESWLQPRDSPVSQLFRRAAPDPNASDYLSHYPSPGSTP  60

H99      NVSTIPQAWLDKLATVNLPNVPVATPDGGRPTYPNNEDDGDSTICSFTDQCRVEDDLYSP  120
         NVSTIPQAWLDKLATV LPNV VAT  G  PTYPNNE+DGDSTICSFTDQC   DDL+SP
R265     NVSTIPQAWLDKLATVQLPNVSVATASGEIPTYPNNENDGDSTICSFTDQCVEPDDLFSP  120

H99      PGEKIWALSFDDGPTDVSPALYDYLAQNNISSSATHFMIGGNVITSPQSVLVAVKAGGHL  180
         PGEKIWALSFDDGPTDVSP LYD+LAQNNISS ATHFMIGGNV+TSPQSVL+AV+AGGHL
R265     PGEKIWALSFDDGPTDVSPGLYDFLAQNNISSKATHFMIGGNVVTSPQSVLIAVQAGGHL  180

H99      AVHTWSHPYMTTLTNEQVVGELGWTMQALSDLNGGRIPMYWRPPYGDVDNRVRAIAKEVF  240
         AVHTWSHPYMTTLTNEQVVGELGWTMQALSDLNGGR+P +WRPPYGDVDNRVRAIAK VF
R265     AVHTWSHPYMTTLTNEQVVGELGWTMQALSDLNGGRVPKFWRPPYGDVDNRVRAIAKGVF  240

H99      GLVTVLWDSDTNDWAITDEPGQYSVASVEAYFDTLVTGNRTQGLLLLEHELDNNTVEVFE  300
          L TVLWD DTNDWAI DEP QYS+ASVEAYFDTLVTGNRTQGLLLLEHELDNNTV VFE
R265     DLETVLWDEDTNDWAIADEPSQYSIASVEAYFDTLVTGNRTQGLLLLEHELDNNTVTVFE  300

H99      TEYPKAVGNGWTVKNVADAFNMEWYLNSGKGNNDVVTTMSVAGTLTTATPTNTSTYVASS  360
         TEYPKA+ NGW VKNVADAFNMEWYLNSGKGN+  VTTMSV GTL TA PTNTST VAS+
R265     TEYPKAIANGWIVKNVADAFNMEWYLNSGKGNDATVTTMSVGGTLPTAAPTNTSTSVASA  360

H99      TAASSASVTDSAGVSIASAASSEASSSWAIANRPSHFVIAIACGLALAAIMV  412
         +A SS SVTDSAGVSIASAASSE+SSSWAIA RPS F+  IACGL  AA +V
R265     SATSSGSVTDSAGVSIASAASSESSSSWAIAERPSLFI--IACGLVFAAAVV  410
```

352/412 identical (85%); 372/412 positive (90%)
