# Supplementary material for: Chitosan Biosynthesis and Virulence in the Human Fungal Pathogen Cryptococcus gattii
Source: mSphere. 2019 Oct 9;4(5):e00644-19. doi: 10.1128/mSphere.00644-19 (PMC6796976; doi:10.1128/mSphere.00644-19)
Supplement: TABLE S1 [file mSphere.00644-19-st001.pdf]

S. Table 1

Primer name	sequence
1-Cda1 (R265)	CGTTTGACTGCGGACTTAG
2-Cda1 (R265)	AGTTCCAACCGAAGACAGG
3-Cda1 (R265)	ACGGTCGCTTTTTAGACGCcaggaaacagctatgaccatg
4-Cda1 (R265)	catggtcatagctgtttcctgGCGTCTAAAAAGCGACCGT
5-Cda1 (R265)	cactggccgtcgttttacaacAGGGCTTTCATTGTAGCG
6-Cda1 (R265)	CGCTACAATGAAAGCCCTgttgtaaaacgacggccagtg
7-Cda1 (R265)	GGAAGGAATCATTGTTCGTTCG
8-Cda1 (R265)	ACAGTCATCAAAGGTTCGG
9-Cda1 (R265)	TGAGCTCGCTCAAAGCGCTAG
10-Cda1 (R265)	TGCTGTTGCTGTTGGTATAGTA
1-Cda2 (R265)	AGTAGTCGGTCAATAATGCG
2-Cda2 (R265)	GGGTTCCAAGACTGATAAGC
3-Cda2 (R265)	CGTTTTACCTTTTCCGCTCcaggaaacagctatgaccatg
4-Cda2 (R265)	catggtcatagctgtttcctgGAGCGGAAAAGGTAAAACG
5-Cda2 (R265)	cactggccgtcgttttacaacGCATACAACATCACCCAACC
6-Cda2 (R265)	GGTTGGGTGATGTTGTATGCgttgtaaaacgacggccagtg
7-Cda2 (R265)	AAATACTAACCGCACTCGC
8-Cda2 (R265)	TATTATCACCCAGCAGTCCTCCCG
9-Cda2 (R265)	TTCGCCTGCGTGATTAAGAG
10-Cda2 (R265)	GTGTTTTCGTGTCGATGTTCC
1-Cda3 (R265)	CGTTCTGTGCCAAAAACC
2-Cda3 (R265)	TGCTGATGCTCTACGAGTGC
3-Cda3 (R265)	CCAGGACTGATTTACACTGTCAcaggaaacagctatgaccatg
4-Cda3 (R265)	catggtcatagctgtttcctgTGACAGTGTAAATCAGTCCTGG
5-Cda3 (R265)	cactggccgtcgttttacaacGAATCATCTTCTTCGTGGG
6-Cda3 (R265)	CCCACGAAGAAGATGATTCgttgtaaaacgacggccagtg
7-Cda3 (R265)	TCCCGAAAGTCAGAAGGTC
8-Cda3 (R265)	CGTCAGCAAATGATAGGTG
9-Cda3 (R265)	GACCGTACATTTTAATAGGC
10-Cda3 (R265)	CCGCCCCAACCAATACTTCT
