# Supplementary figures and images for: Chitosan Biosynthesis and Virulence in the Human Fungal Pathogen Cryptococcus gattii
Source: mSphere. 2019 Oct 9;4(5):e00644-19. doi: 10.1128/mSphere.00644-19 (PMC6796976; doi:10.1128/mSphere.00644-19)

## S. Fig.2

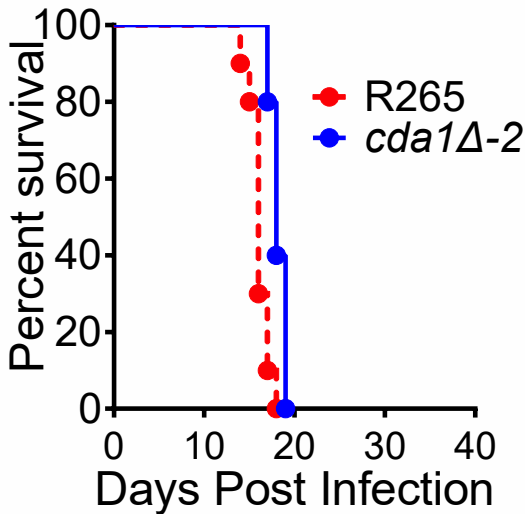

Supplement: FIG S2 [file mSphere.00644-19-sf002.pdf]

## S. Fig. 3

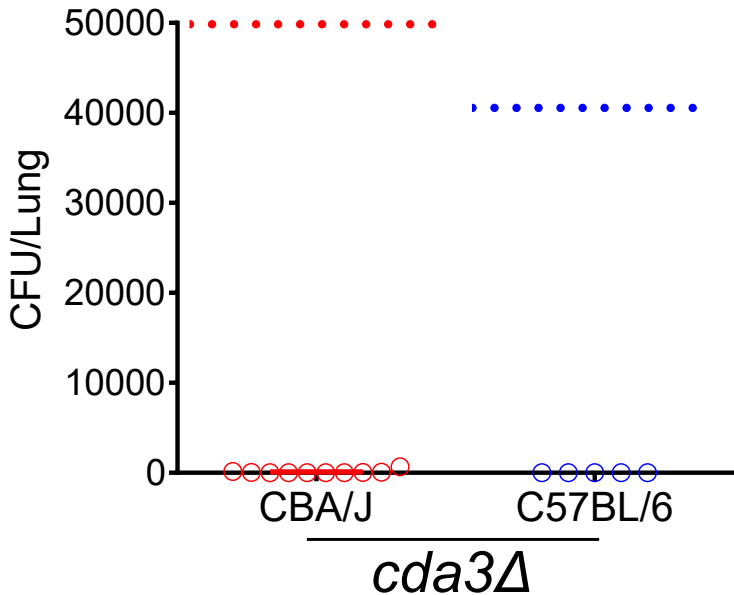

Supplement: FIG S3 [file mSphere.00644-19-sf003.pdf]

# S. Fig. 4

## A

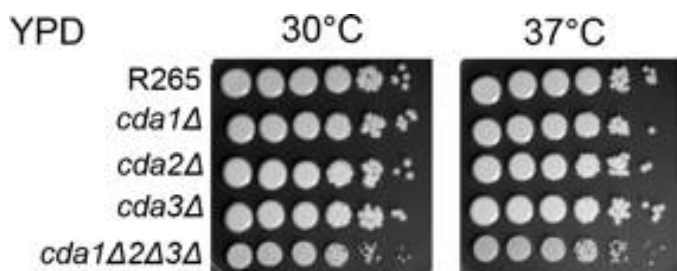

## B

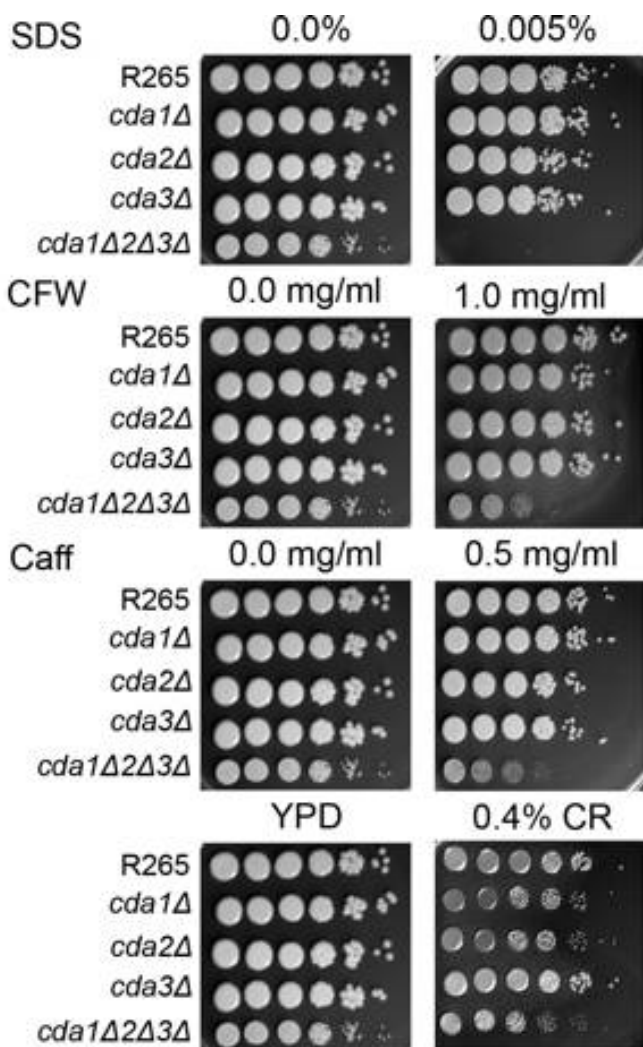

Supplement: FIG S4 [file mSphere.00644-19-sf004.pdf]

# S. Fig. 5

A

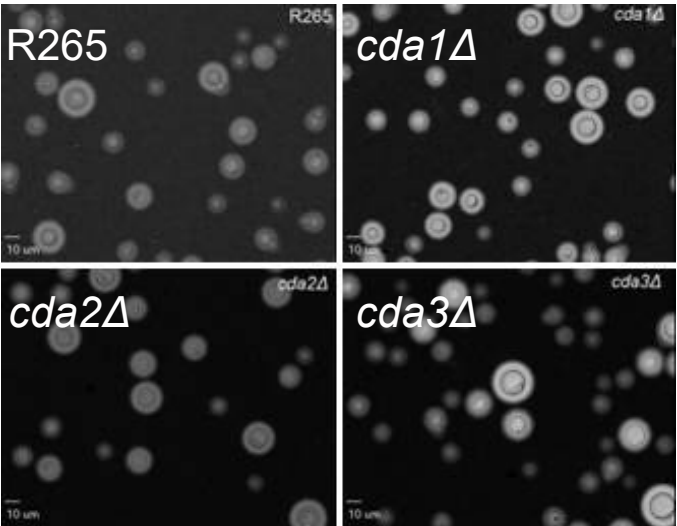

B

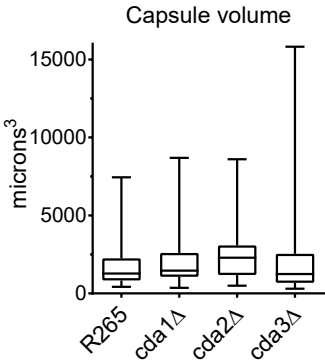

C

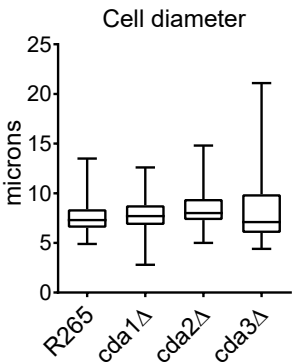

Supplement: FIG S5 [file mSphere.00644-19-sf005.pdf]

# S. Fig. 6

## A

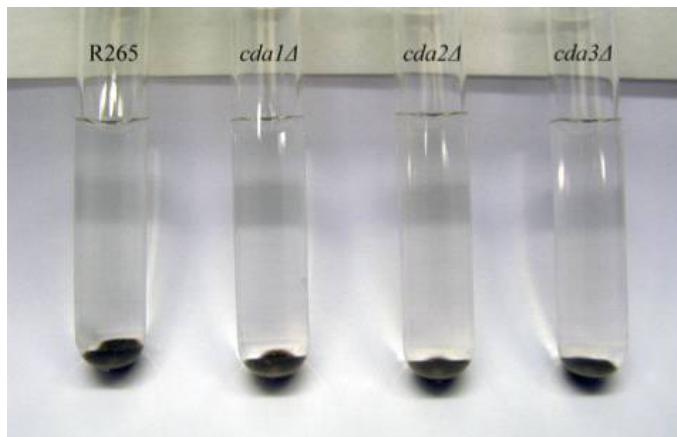

## B

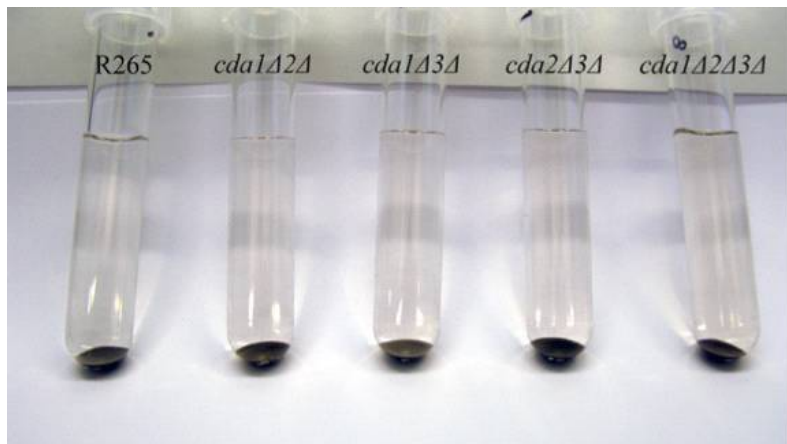

Supplement: FIG S6 [file mSphere.00644-19-sf006.pdf]

## S. Fig. 7

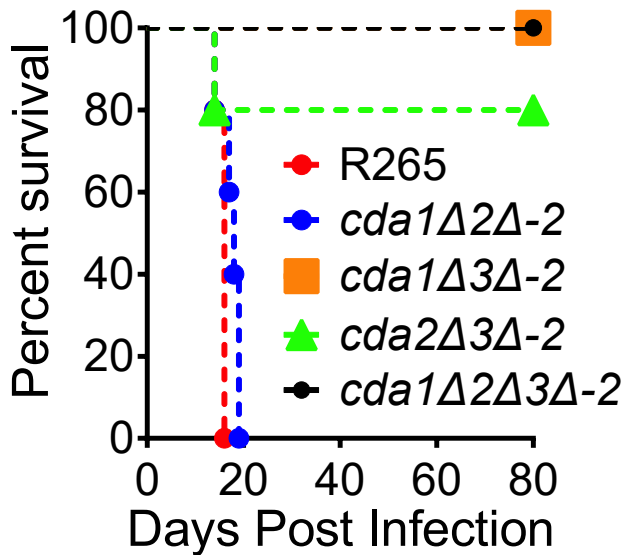

Supplement: FIG S7 [file mSphere.00644-19-sf007.pdf]

## S. Fig. 8

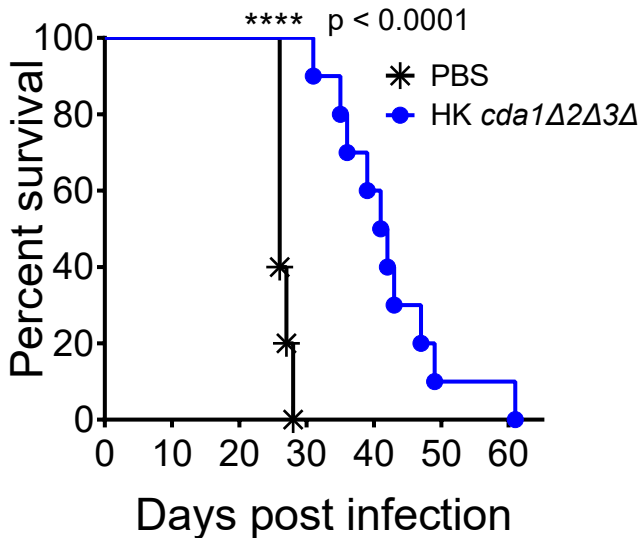

Supplement: FIG S8 [file mSphere.00644-19-sf008.pdf]
